# Stimuli-Responsive Poly[oligo(ethylene glycol) methacrylate] Monolayers: Reversible Temperature-Driven Swelling Dynamics, Tunable LCST, and Antifouling Properties

**DOI:** 10.1021/acsapm.6c00888

**Published:** 2026-05-14

**Authors:** Silvija Juciute, Egle Ezerskyte, Kristina Bolgova, Medeina Steponaviciute, Emile Peciukaityte, Ieva Plikusiene, Vaidas Klimkevicius

**Affiliations:** † Institute of Chemistry, Faculty of Chemistry and Geosciences, 54694 Vilnius University, Naugarduko strasse 24, LT-03225 Vilnius, Lithuania; ‡ Biomedical Physics Laboratory, National Cancer Institute, Baublio strasse 3b, LT-08406 Vilnius, Lithuania; § Institute of Biosciences, Life Sciences Center, Sauletekio avenue 7, LT- 10257 Vilnius, Lithuania; ∥ State Research Institute, Centre for Physical and Technological Sciences, Sauletekio avenue 3, LT-10257 Vilnius, Lithuania

**Keywords:** OEGMA-based copolymers, RAFT polymerization, surface immobilization, thermally induced phase transition, protein adsorption resistance, biointerface engineering

## Abstract

Stimuli-responsive polymers, which can reversibly change their physicochemical properties in response to external stimuli, have broad potential applications in biomedical and surface engineering. Here, we report the synthesis of hydrophilic poly­[oligo­(ethylene glycol) methacrylate] [p­(DEGMA-*co*-OEG_5_MA)] copolymers with tunable lower critical solution temperatures (LCSTs). Copolymers with LCST values near physiological temperature were synthesized via controlled radical polymerization, end-functionalized with thiol groups, and immobilized onto gold substrates to form thermoresponsive monolayers. Real-time quartz crystal microbalance with dissipation (QCM-D) and spectroscopic ellipsometry measurements revealed reversible temperature-dependent swelling and contraction of the monolayers. To the best of our knowledge, this is the first demonstration of the direct formation of hydrophilic p­(DEGMA-*co*-OEG_5_MA) monolayers on gold QCM-D sensors. The monolayers exhibited excellent protein-repelling properties against bovine serum albumin, confirming their antifouling behavior. These findings demonstrate a controllable strategy for fabricating biocompatible, thermoresponsive, and antifouling polymer interfaces with strong potential for biosensing, medical device coatings, and controlled drug delivery applications.

## Graphical abstract

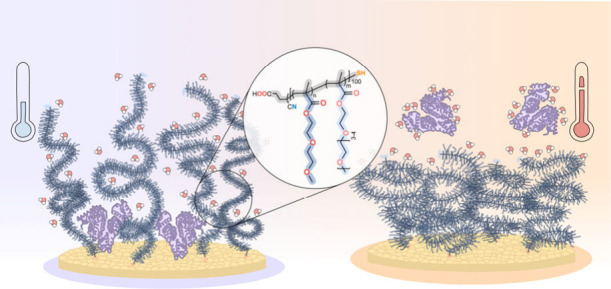

## Introduction

Stimuli-responsive polymers, also known as “smart” materials, are able to change their physical and/or chemical properties in response to external stimuli in their surroundings, e.g., chemical (pH value, ionic strength, solvent),^1–3^ physical (temperature, light, mechanical forces, magnetic fields),^1,4,5^ or even biological (bacteria, biomolecules).^6,7^ Most of these polymers are synthesized by free-radical or living radical polymerization of stimuli-responsive monomer units, or by postpolymerization modification that incorporates responsive chemical functionality into the polymer chain. The responsivity to the stimulus in the polymer is most often revealed as a change in the polymer conformation, and all these changes depend on its structure, chemical composition, and functional groups.^8–10^

In recent decades, numerous stimuli-responsive polymers and copolymers have been synthesized and investigated because of their unique properties (stimuli responsivity).^1,11,12^ Among these materials, most attention is directed toward thermoresponsive polymers. Thermoresponsive polymers are characterized by two temperatures: the lower critical solution temperature (LCST), below which a polymer solution is clear and homogeneous, while a polymer solution above the LCST appears cloudy (cloud point), and the upper critical solution temperature (UCST), above which the polymer solution returns to a clear and homogeneous state. LCST and UCST are the temperature points where the polymer and solvent are fully miscible, with LCST being the lower and UCST the upper limit. Thermoresponsive polymers, which pose reversible solubility change within a slight temperature change, show either a LCST, an UCST, or, in some cases, both. What has sparked interest in them is the potential of inducing their response *in vivo* using noninvasive means, which is particularly important in order to use them for biomedical applications.^9,13^ In such cases, temperature variations in the human body (e.g., differences in the temperature of inflamed/infected tissue) can serve as the stimulus. Thermoresponsive polymers have found various applications in tissue engineering, material separation, multifunctional coatings, self-healing materials, and biosensors, and they are especially important in controlled drug delivery systems.^14,15^

Building on these application stimuli-responsive polymers have been extensively studied in bulk and solution phases, as well as at solid interfaces.^16–20^ In particular, surface-grafted thermoresponsive polymer brushes have attracted considerable scientific interest because they enable precise control over interfacial properties such as wettability, adhesion, lubrication, and biointeractions. These systems have provided significant insight into interfacial swelling behavior, collapse transitions, conformational changes, and responsiveness to external stimuli, including temperature, pH, and ionic strength. Thermoresponsive surface modifications using polymers such as poly­(*N*-isopropylacrylamide) (pNIPAM) have been reported previously.^21,22^ However, to the best of our knowledge, this study represents the first instance of directly forming hydrophilic poly­[oligo­(ethylene glycol) methacrylate] [p­(DEGMA-*co*-OEG_5_MA)] monolayers on gold QCM-D sensors. One major challenge in this field has been the biocompatibility of commonly used polymers such as pNIPAM. Although pNIPAM is well-known for its sharp and reversible thermoresponsive behavior near physiological temperature (LCST ∼ 32 °C),^23,24^ concerns about its potential carcinogenicity, protein adsorption due to secondary amide groups, and limited long-term reversibility have restricted its use in biomedical applications.^25,26^ As a promising alternative, oligo­(ethylene glycol) methyl ether methacrylate (OEGMA)-based copolymers have attracted considerable attention. These polymers are nontoxic, nonimmunogenic, and biocompatible, exhibiting excellent solubility in a wide range of solvents. Importantly, they also exhibit protein-repelling properties, which makes them particularly suitable for biointerface applications where minimizing nonspecific protein adsorption is critical.^16,27–29^ The thermoresponsive behavior of OEGMA-based copolymers can be precisely tuned by varying their hydrophilic–hydrophobic balance. Unlike ionic systems, their response is pH-independent, making them ideal candidates for constructing stimuli-responsive monolayers intended for biomedical use. To better understand and optimize the functionality of such monolayers, advanced surface-sensitive techniques such as quartz crystal microbalance with dissipation (QCM-D) and ellipsometry are crucial. These techniques provide real-time, noninvasive insights into mass uptake, swelling behavior, and conformational changes of polymer layers in response to stimuli. The continued advancement and application of these tools are essential for developing next-generation smart interfaces based on biocompatible, protein-resistant polymers.

Therefore, in this study, we report the synthesis of p­(DEGMA-*co*-OEG_5_MA) copolymers by varying the initial molar ratio of monomers in the reaction feed. The copolymers were characterized to determine their macromolecular parameters, and the effect of composition on the LCST was systematically investigated using dynamic light scattering (DLS). Selected copolymers exhibiting LCST values close to physiological temperature were modified to obtain -SH terminal groups and subsequently immobilized onto gold surfaces to form thermoresponsive monolayers. The temperature-dependent behavior of these monolayers was evaluated using QCM-D and ellipsometry. Furthermore, a comprehensive analysis of the protein-repelling properties of the thermoresponsive polymer-modified and bare gold surfaces was conducted upon exposure to bovine serum albumin (BSA).

## Materials and Methods

### Materials

Di­(ethylene glycol) monomethyl ether methacrylate (DEGMA, *M*_n_ = 188.22, Aldrich) and oligo­(ethylene glycol) monomethyl ether methacrylate (OEGMA, *M*_n_ = 300, Aldrich) were purified from inhibitors by passing through a chromatographic column filled with basic alumina (Type 5016A, Fluka). 1,4-Dioxane (DO, 99.8%, Eurochemicals) and thermal initiator 4,4-azobis­(4-cyanovaleric acid) (ACVA, 98%, Fluka) were used as received without further purification. Reversible addition–fragmentation chain-transfer (RAFT) agent (CTA) 4-[[(butylthio)­carbonothioyl]­thio]-4-cyanopentanoic acid was synthesized prior to polymerization according to a previously published procedure.^30^ For QCM-D and ellipsometry measurements, QCM-D gold-covered sensor disks were purchased from Biolin Scientific. Protein-repelling properties were evaluated using bovine serum albumin (BSA, Carl Roth).

### RAFT Polymerization of Thermoresponsive p­(DEGMA-*co*-OEG_5_MA) Copolymers

RAFT copolymerization of DEGMA and OEG_5_MA monomers at various initial molar ratios ([DEGMA]_0_:[OEG_5_MA]_0_) = 100:0; 90:10; 85:15; 80:20; 75:25; 70:30; 65:35; 60:40; 50:50; 40:60; 30:70; 20:80; 10:90; 0:100) was carried out in DO. The initial molar ratio of total monomer to the CTA and the initiator was kept constant at [M]_0_:[CTA]_0_:[ACVA]_0_ = 300:3:1. The total concentration of reagents in solution was 15 wt %. All copolymers were synthesized under identical polymerization conditions.

A more detailed description of the synthesis of the p­(DEGMA-*co*-OEG_5_MA) with an initial monomer ratio of 80:20 is provided below.

DEGMA (1.476 mL, 1.506 g, 8 mmol), OEG_5_MA (0.571 mL, 0.6 g, 2 mmol), CTA (29.1 mg, 0.1 mmol), and ACVA (9.3 mg, 0.033 mmol) were added to a 25 mL round-bottom flask and dissolved in 14.04 mL of DO. The reaction mixture was degassed by bubbling nitrogen gas for 20 min and then stirred magnetically at 70 °C for 24 h. The reaction was quenched by cooling the flask to room temperature and exposing it to air.

The resulting copolymers were purified by dialysis against deionized (DI) water using a 3.5 kDa molecular weight cutoff membrane, followed by freeze-drying. The products were obtained as yellowish viscous oils. Yields were determined gravimetrically and ranged from 86 to 95%.

### Aminolysis of the Terminal Trithiocarbonate Groups of the Synthesized Copolymers

Aminolysis of the terminal trithiocarbonate groups of the synthesized copolymers to obtain −SH terminal groups was carried out according to a published procedure.^31^ Briefly, 0.5 g of p­(DEGMA-*co*-OEG_5_MA) was dissolved in 10 mL of DI water, followed by the addition of an excess of hydrazine monohydrate (0.2 mL). The mixture was stirred vigorously for 20 min at room temperature. Afterward, the products were purified by dialysis against DI water using a 3.5 kDa molecular weight cutoff membrane and isolated by freeze-drying.

### Proton Nuclear Magnetic Resonance (^1^H NMR) Spectroscopy

All ^1^H and ^13^C NMR spectra were recorded on a Bruker 400 Ascend nuclear magnetic resonance spectrometer (400 MHz) in DMSO-*d*_6_ at 22 °C.

### Dynamic Light Scattering (DLS)

DLS measurements were performed using a Zetasizer Nano ZS (Malvern Instruments, Malvern) equipped with a 4 mW He–Ne laser operating at 633 nm. Samples were prepared as 1% (w/w) solutions in distilled water. Measurements of scattered light intensity were performed at an angle of 173°. The measurements were conducted over a temperature range of 0–93 °C. The samples were allowed to equilibrate for 5 min before each measurement. During the temperature mode, the samples were measured at 1 °C increments for better accuracy. The size distributions were obtained from the correlation functions, and the data were analyzed using the Malvern Zetasizer software v. 7.03.

### Size-Exclusion Chromatography (SEC)

Different macromolecular parameters of copolymers, such as number-average and weight-average molecular weights (*M*_n_ and *M*_w_) and dispersity (*Đ* = *M*_w_/*M*_n_), were determined by SEC. SEC measurements were carried out in sodium acetate buffer (250 mM, pH 4.0) and THF as the eluent at 30 °C with a flow rate of 0.5 mL/min. Viscotek (Malvern) columns AGuard (50 × 8.0 mm) and A6000 M General Mixed Aq (300 × 8.0 mm) were used. A Viscotek TDAmax (Malvern) system equipped with a triple detection array (TDA305) consisting of a refractive index (RI) detector, light scattering (LS) detector, simultaneously measuring the scattered light (laser 3 mW, 670 nm) at two angles [right-angle (90°) and low-angle (7°)], and a four-capillary bridge viscosity detector was used. The system was calibrated using PEO standards for triple calibration PolyCAL TDS-PEO-N (*M*_w_ = 24 kDa, Malvern). SEC data were collected and processed using OmniSEC software (Malvern, v. 5.12).

### Quartz Crystal Microbalance with Dissipation (QCM-D) and Spectroscopic Ellipsometry (SE) Combination

QCM-D measurements were performed using a QSense Explorer (Biolin Scientific, Sweden). It operates at a fundamental frequency of 5 MHz and simultaneously registers up to 7 harmonics. Collected data were analyzed with QSense DFind software (1.2.7). For SE/QCM-D combined measurements, a QCM-D measurement module with optical windows was mounted on a table for ellipsometry (chamber volume ∼ 100 μL). A spectroscopic ellipsometer with a rotating compensator, the M-2000X (J. A. Woolam, USA), was used. The incidence angle was fixed at 65°, and the signal was recorded over the spectral range 200–1000 nm. Fluids were injected at a fixed 1 mL/min speed with an Ismatec IPC4 peristaltic pump (Cole-Palmer GmbH, Germany). Measurements were performed under static conditions; after the fluid was injected, the pump and thus the flow were stopped.

### Atomic Force Microscopy (AFM) Surface Analysis

Surface morphology and roughness of dried samples were investigated using an atomic force microscope (diInnova, Veeco Instruments Inc., USA). Measurements were performed in ambient air under tapping mode using TESPA-V2 probes (Bruker, USA) with a nominal resonance frequency of *f*_0_ = 320 kHz and a spring constant of *k* = 42 N/m.

AFM scans were acquired at randomly selected locations on each sample to ensure representative surface characterization. Topographic images were recorded over scan areas of 5 × 5 μm^2^, 2 × 2 μm^2^, and 1 × 1 μm^2^, with a resolution of 512 × 512 pixels.

The acquired AFM images were processed using SPMLabAnalysis V7.00 (Build R1.44874) software (Veeco Instruments Inc., USA). Image processing included plane correction (flattening) to eliminate sample tilt and scanner-induced artifacts. A second-order horizontal flattening procedure was applied to all images prior to analysis. Surface roughness was quantified using the software’s *Area Analysis → Surface Roughness function*. The root-mean-square (RMS) roughness (*R*_s_ or *R*_q_) values were extracted from the flattened images and used for quantitative comparison.

### Polymer Immobilization

Before polymer immobilization, the QCM-D sensor disk was washed in an ultrasonic bath with methanol for 2 min, followed by air drying. Subsequently, the QCM-D sensor disk was equilibrated in DI water at 20 °C. Following this, a polymer solution at 10 mg/mL in DI water was introduced into the measurement chamber and maintained for 4 h to ensure steady-state conditions. Each polymer immobilization procedure was subsequently complemented by a DI water wash to remove any unbound polymers.

### Thermoresponsiveness of the Polymeric Monolayer on the QCM-D Sensor Disk

After successfully immobilizing the polymer, a heating–cooling cycle was conducted. The temperature was gradually increased from 20 to 50 °C and subsequently reduced back to 20 °C. The temperature was adjusted in increments of 2 °C, with a 10 min equilibration period at each step to ensure stability before measuring the polymer’s response.

### Protein-Repelling Properties

In order to evaluate the protein-repelling properties of the polymer layer, BSA was selected as a test protein. Experimental investigations were conducted at two distinct temperatures, 20 and 40 °C. At 20 °C, following the formation of the polymer layer, a 500 nM solution of BSA in DI water (pH = 7.4) was introduced into the measurement chamber and incubated for 30 min. For experiments conducted at 40 °C, a fresh polymer layer was immobilized, and the measurement chamber temperature was subsequently elevated to 40 °C. Upon reaching thermal equilibrium, a 500 nM BSA solution was injected into the chamber for an additional 30 min. For comparative analysis, interactions between BSA and a nonmodified gold QCM-D sensor disk were also monitored under both temperatures, serving as a reference for the experimental results.

## Results and Discussion

### Synthesis and Investigation of the Composition-Dependent Thermoresponsive Properties of p­(DEGMA-*co*-OEG_5_MA) Copolymers

The copolymers were synthesized through a controlled RAFT polymerization process, as outlined in the Materials and Methods section. Following the synthesis, the potentially toxic CTA end groups were removed by treating the aqueous polymer solution with hydrazine (Figure 1a). This treatment resulted in a product that was both colorless and odorless. Modifying the end-group functionality of the synthesized copolymers offers multiple advantages. First, the removal of hydrolysis-sensitive trithiocarbonate groups mitigates the risk of generating volatile sulfur compounds during applications, which are not only toxic but also associated with unpleasant odors. Second, the introduction of −SH terminal groups enhances the potential for further functionalization of the polymers and improves their adsorption properties, particularly on surfaces such as those coated with gold.^32,33^ The precise compositions of the p­(DEGMA-*co*-OEG_5_MA) copolymers were calculated from ^1^H NMR spectra using the equation shown in Figure S1.

**Figure 1:**
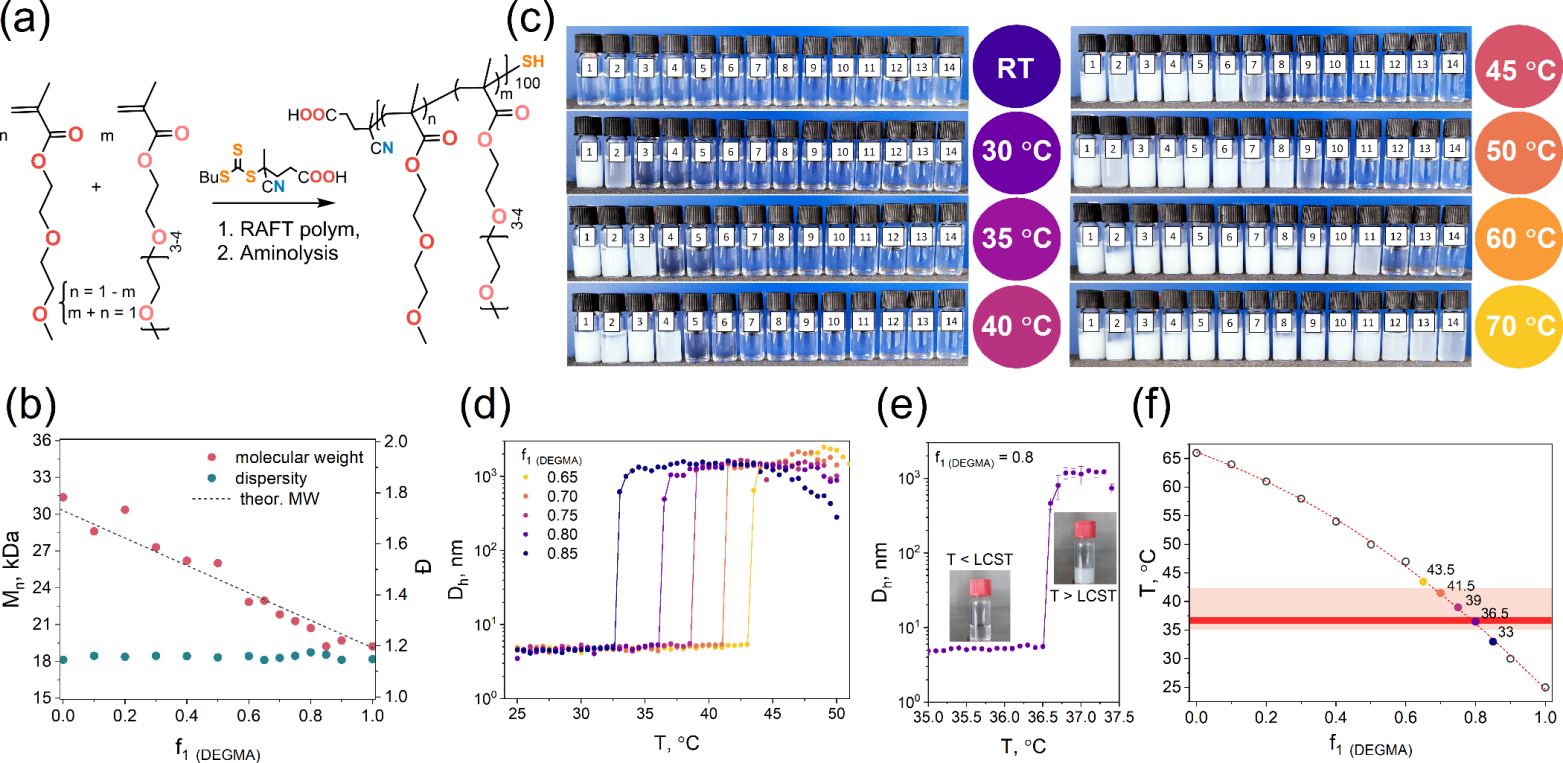
Synthesis of p­(DEGMA-*co*-OEG_5_MA) copolymers with varying compositions (a). The macromolecular parameters, including molecular weight and dispersity, of the synthesized copolymers are analyzed as a function of the initial monomer feed (*f*_1 (DEGMA)_) (b). Visual turbidity of thermoresponsive copolymers as a function of temperature (c). Monomer compositions *f*_1(DEGMA)_: 1 (1); 0.9 (2); 0.85 (3); 0.8 (4); 0.75 (5); 0.7 (6); 0.65 (7); 0.6 (8); 0.5 (9); 0.4 (10); 0.3 (11); 0.2 (12); 0.1 (13); 0 (14). Change in the DLS hydrodynamic diameter in relation to the temperature for samples exhibiting LCST near the biological temperature range, with measurements recorded at intervals of 0.5 °C (d). A precise LCST determination for the p­(DEGMA_0.8_-*co*-OEG_5_MA_0.2_) sample, along with a visual representation of the samples changing from clear to cloudy as the LCST is reached (e). LCST of the synthesized copolymers as a function of the DEGMA compositions (*f*_1(DEGMA)_) (f). The bright red line in part d represents the human physiological temperature.

The results for *M*_n_ and dispersity (*Đ*) of the synthesized copolymers are shown in Figure 1b. As anticipated, it was observed that the molecular weight (*M*_n_) decreases with an increasing molar fraction of DEGMA monomeric units within the composition. For the majority of the samples analyzed, the molecular weights of copolymers closely match the theoretical values, suggesting an exceptionally high monomer conversion. Moreover, the copolymers exhibited a narrow molecular weight distribution (*Đ* < 1.2), indicating the successful, controlled nature of the RAFT polymerization.

The copolymers were further functionalized by treating their aqueous solutions with hydrazine. Hydrazine proves to be particularly effective in removing CTA groups while minimizing the formation of disulfides, which can occur when using alternative reagents for this removal process.^34–36^ However, this method required additional purification of the polymers; therefore, the polymers were subsequently purified by dialysis against DI water prior to further use. The molecular weight distribution curves of the p­(DEGMA-*co*-OEG_5_MA (*f*_1(DEGMA)_ = 0.8) sample, both before and after aminolysis, exhibited a unimodal character (please refer to Figure S3a). This indicates that no disulfide bonds were formed, which suggests that there was no significant doubling in the molecular weight of the copolymers. This was also confirmed by recording SEC-RI eluograms; neither did the curve show a shift toward lower retention volumes, nor was there any shoulder observed in the elution peak (Figure S3b). The molecular weights of the CTA- and HS-terminal samples were virtually identical, at approximately 21.2 and 21.5 kDa, respectively. However, the lack of change in the copolymer’s molecular weight throughout the treatment does not necessarily confirm successful functionalization. Consequently, the SEC-UV eluograms were recorded and presented in Figure S3c. The SEC-UV reveals a different aspect of the analysis; it is evident that the UV absorption peak at 310 nm, associated with the terminal CTA groups in the copolymer, experienced a significant reduction. After aminolysis, the area under this peak decreased from 459 mV·mL to as low as 7.1 mV·mL, indicating that 98.5% of the terminal CTA groups were converted to thiol.

Terminal thiol groups are beneficial for modifying gold-coated surfaces due to their ability to form strong and stable bonds with gold, resulting in robust surface attachment. However, before creating thermoresponsive monolayers on gold surfaces, it is important to evaluate the conformational changes of the p­(DEGMA-*co*-OEG_5_MA) copolymer in aqueous solutions as a function of increasing temperature, considering different compositions.

First, the visual changes in transparency of the prepared 1% (w/w) aqueous solution of p­(DEGMA-*co*-OEG_5_MA) with different content of DEGMA monomeric units in composition were evaluated as a function of increased temperature (Figure 1c). Obviously, all the samples were clear at room temperature (20 °C), and cloudy when the temperature reached/exceeded 70 °C. The cloud points of the samples were related to copolymer composition, with the most promising being p­(DEGMA-*co*-OEG_5_MA), composed of a relatively high content of DEGMA monomeric units (*f*_1(DEGMA)_ = 0.65–0.85). For such samples, the visual transmission of the clear sample changed in the temperature range between 30 and 45 °C (Figure 1c, samples 2–7). It is noteworthy that the transition of the solution from a clear to a turbid state is fully reversible and is unaffected by the number of heating and cooling cycles. The precise LCST of the aforementioned copolymer samples was determined by monitoring changes in their hydrodynamic diameter using DLS in aqueous solution, as presented in Figure 1d. DLS measurements were conducted at 0.5 °C intervals over the temperature range of 25–50 °C, with a temperature stabilization period of 2 min at each step. Below the LCST, all investigated copolymers exist as individual random coils with hydrodynamic diameters smaller than 10 nm. Upon reaching the LCST, a sharp increase in the hydrodynamic diameter (*D*_h_) to 10^3^ nm was observed, indicating the formation of polymer aggregates as a result of chain dehydration. This transition is attributed to the disruption of hydrogen bonding between the polymer chains and surrounding water molecules, thereby enhancing hydrophobic interactions among the oligo­(ethylene glycol) side chains. Similar findings have been reported by Ramírez-Jiménez et al.^37^

It was observed that the LCST of the p­(DEGMA-*co*-OEG_5_MA) copolymer, containing approximately 80 mol % DEGMA, closely aligns with human physiological temperature. The clear-to-cloudy phase transition occurred between 36 and 37 °C. Due to the narrow transition range, additional measurements were performed with high precision. Specifically, the *D*_h_ of the copolymer was recorded as a function of temperature at 0.1 °C intervals within the 35–37.5 °C range, using DLS. A 5 min temperature stabilization period was maintained at each measurement point to ensure thermal equilibrium (Figure 1e). The LCST values of all investigated copolymers are summarized in Figure 1f. A general trend of decreasing LCST with increasing DEGMA molar content was observed. However, unlike previously reported data in the literature^37^, this relationship could not be accurately described by linear extrapolation. The discrepancy may be attributed to the more precise determination of copolymer composition in the present study.

### Formation and Study of a Thermoresponsive Protein-Repelling Monolayer on Gold Surfaces

Polymer monolayers with thiol (−SH) functional groups were formed directly on the gold-coated QCM-D sensor disks. The visual illustration of possible conformational changes in the thermoresponsive copolymer during immobilization, and under applied/removed stimuli, is depicted in Figure 2a. The immobilization process was monitored in real time using a combination of QCM-D and SE. A 10 mg/mL aqueous polymer solution was injected into the measurement chamber and allowed to react for 4 h. After the steady state was reached, the chamber was rinsed with water to remove loosely bound and freely floating polymer chains.

**Figure 2:**
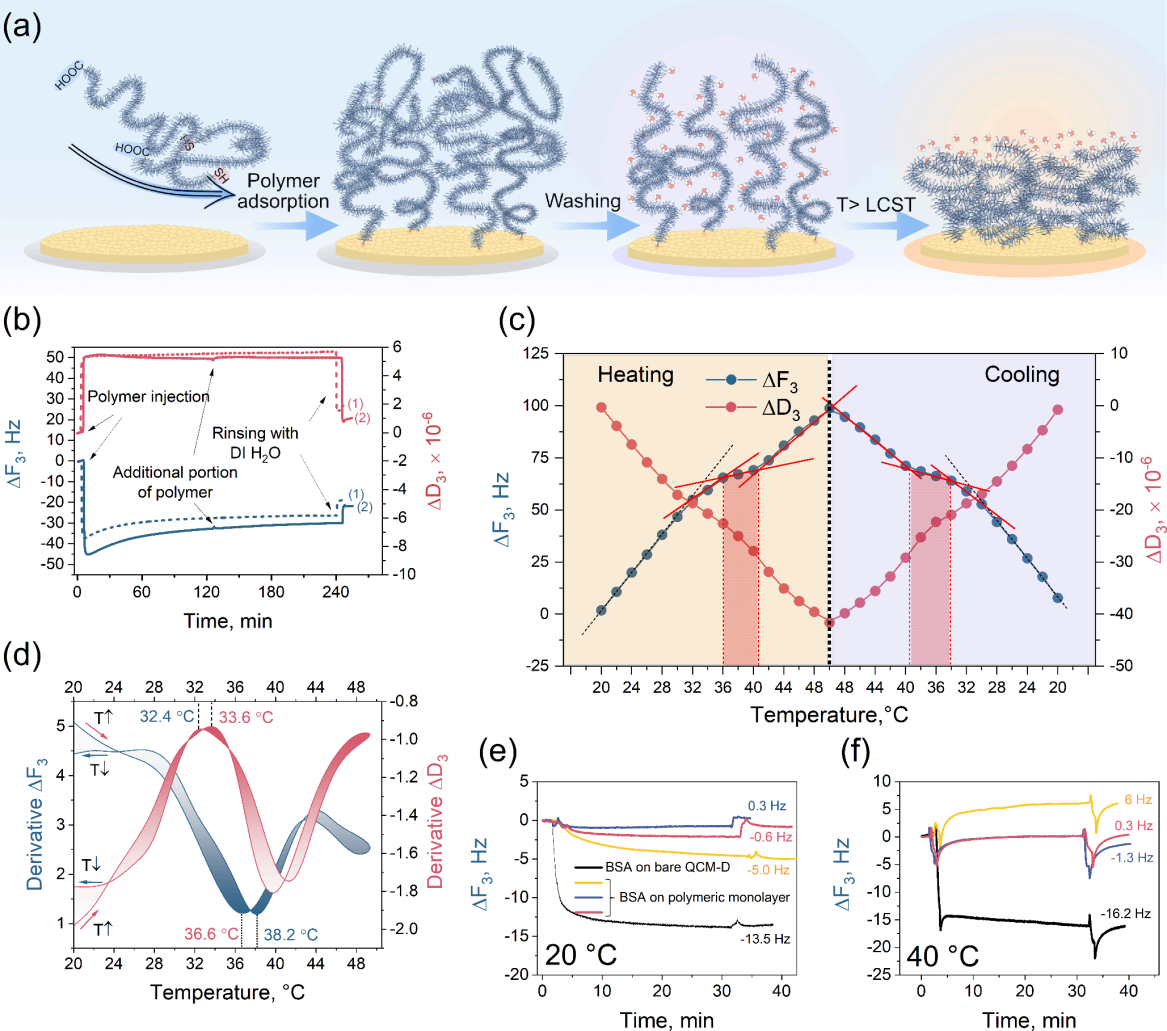
Visual summary of the thermoresponsive p­(DEGMA-*co*-OEG_5_MA) monolayer formation on QCM-D gold surfaces with possible conformational changes of the copolymer under applied/removed stimuli (a). Kinetics of polymer monolayer formation [two individual repetitions labeled as (1) and (2)]: evolution of Δ*F* and Δ*D* over time (b). Changes in Δ*F* and Δ*D* during a heating–cooling cycle (c). Derivative curves of Δ*F* and Δ*D* with labeled extrema (d). BSA adsorption on bare (black curves) and polymer-modified QCM-D sensors at 20 °C (e) and 40 °C (f). Yellow curves indicate the first adsorption on modified surfaces, while blue and red curves show BSA adsorption on surfaces with previously adsorbed BSA.

The QCM-D data presented in Figure 2b show the evolution of the resonance frequency (Δ*F*) and dissipation (Δ*D*) during the surface modification with thiol-terminated p­(DEGMA-*co*-OEG_5_MA), a polymer exhibiting a LCST close to physiological temperature (36.5 °C). Changes in Δ*F* reflect variations in the coupled mass at the sensing surface; a decrease in Δ*F* corresponds to an increase in the deposited material, including associated water molecules. However, Δ*F* alone does not provide a direct measure of the adsorbed mass, and quantitative analysis requires the application of appropriate viscoelastic models. In parallel, Δ*D* provides insight into the viscoelastic properties of the formed layer. Low Δ*D* values indicate the formation of rigid, compact films with minimal energy dissipation, whereas higher Δ*D* values suggest softer, more hydrated, and viscoelastic layers. For clarity, only the third overtone is shown in the main text, while the complete overtone data are provided in Figure S5.

Before the rinsing step, the first immobilization repetition (1) (Figure 2b) showed a frequency shift (Δ*F*) and dissipation change (Δ*D*) for the third overtone, which were −26.3 Hz and 5.71 × 10^–6^, respectively. Washing the chamber with water increased the frequency by 7.4 Hz and decreased the dissipation by 3.73 × 10^–6^, indicating removal of loosely bound and weakly adsorbed polymer chains. Overall, formation of a stable polymer monolayer on gold resulted in net changes of −18.9 Hz in Δ*F* and 1.98 × 10^–6^ in Δ*D*. These values suggest the formation of a relatively compact layer with moderate viscoelastic character. To test the reproducibility of surface modification, the immobilization experiment was repeated [curves marked (2) in Figure 2b]. For the second repetition, the decrease in Δ*F* was slightly higher (−32.8 Hz) compared to the first repetition (−27.6 Hz) after 2 h of polymer injection. Thus, it was decided to check whether surface coverage could be further increased; an additional portion of polymer solution was injected into the chamber. As shown in Figure 2b (blue curve), no additional decrease in Δ*F* (i.e., no further increase in adsorbed mass) was observed immediately after the second injection of polymer solution. After two more hours, the chamber was rinsed with DI water. At this point, the net Δ*F* was −21.7 Hz compared to −18.9 Hz of the first repetition, corresponding to a modest additional adsorption of approximately 2.9 Hz. The presented results reveal that initial polymer injection is sufficient to achieve copolymer saturation on the QCM-D gold surface, and a second polymer injection is unnecessary because the gold surface has limited or no additional binding sites (area) available for adsorption. The successful formation of the polymer monolayer was independently confirmed by the optical SE method (please refer to Figure S6). After steady state was reached, the ellipsometry parameter Δ increased by 0.7°. Applying an optical model to the SE data yielded a layer thickness of 20.1 nm (for a comprehensive overview of the optical model, please refer to the Supporting Information), which is in good agreement with the estimated contour length of the polymer chains and does not exceed the maximum theoretical extension of the macromolecules.

In addition, dry (Γ^SE^) and wet (Γ^QCM‑D^) surface mass densities of the polymer monolayer were determined. SE, being highly sensitive to thin films, was used to calculate the dry surface mass density (Γ^SE^), excluding water entrapped in the monolayer, using the de Fejter equation ([Disp-formula eq1]).

1 $$\Gamma^{\mathrm{SE}}=\frac{d\left(n_{\mathrm{layer}}-n_{\mathrm{water}}\right)}{dn/dc}\times 100$$

Here, *d* is the thickness of the polymer layer (20.1 nm), d*n*/d*c* is the refractive index increment [for p­(DEGMA-*co*-OEG_5_MA) copolymer, it is estimated to be 0.132], and *n*_layer_ and *n*_water_ are the refractive indices of the polymer layer and water, respectively.^38^

The wet surface mass density (Γ^QCM‑D^), which includes contributions from both the polymer molecules and the surrounding water, was determined from QCM-D measurements. The polymer monolayer exhibited viscoelastic properties, as indicated by a density change (Δ*D*) of 1.98 × 10^–6^. To analyze this behavior, we used the SmartFit model, which is derived from the Voinova–Voigt viscoelasticmodel.^39^ The calculated Γ^QCM‑D^ was 1357 ng/cm^2^, which is 13.4 times higher than the dry mass density Γ^SE^ (101 ng/cm^2^).

The two independent values (Γ^SE^ and Γ^QCM‑D^) were then used to estimate the hydration of the formed layer using the equation.^40^

2 $$f_{\mathrm{PBS}}=1-\frac{\Gamma^{\mathrm{SE}}}{\Gamma^{\mathrm{QCM‐D}}}$$

The calculated hydration fraction was 89.6%, in excellent agreement with the SE optical model, which estimated a water content of 88.1%. This close correspondence between the two methods strongly confirms both the reliability of the optical modeling and the highly hydrated nature of the polymer monolayer.

After successful formation of a p­(DEGMA-*co*-OEG_5_MA) monolayer, the thermoresponsive behavior of the modified surface was evaluated using temperature ramps across the LCST of the copolymer (36.5 °C, determined by DLS, please refer to Figure 1f). The QCM-D measurement chamber enabled real-time monitoring of layer properties during heating and cooling cycles. Heating was initiated at 20 °C, and the temperature was increased in 2 °C increments every 10 min until reaching a final temperature of 50 °C. The cooling cycle followed the same protocol, with the temperature decreased by 2 °C every 10 min until it reached 20 °C. Throughout the cycles, we recorded changes in frequency (Δ*F*) and dissipation (Δ*D*), as illustrated in Figure 2c. Data points were collected immediately prior to each temperature change, and the variations in Δ*F* and Δ*D* over time during the heating–cooling cycle are shown in Figure S7. Because the resonance frequency is sensitive to temperature fluctuations, control heating experiments were performed using a bare gold sensor, and the results are presented in Figure S8.

As expected, the applied temperature caused partial collapse of the polymer layer, resulting in an increase in frequency (Δ*F*) due to loss of coupled water and a simultaneous decrease in dissipation (Δ*D*), reflecting stiffening of the layer. Cooling, on the other hand, restored hydration, indicated by a decrease in Δ*F* and an increase in Δ*D*, confirming reversible swelling behavior.

At the beginning of the heating process, a rapid increase in Δ*F* was observed (from 20 to 30 °C). When the temperature exceeds 30 °C, the tendency to increase in Δ*F* becomes nonlinear. The slowest increase in Δ*F* is observed in a range between 35 and 40 °C. Above 40 °C, Δ*F* continues to increase gradually. During cooling, a similar pattern was observed: Δ*F* initially decreases, then plateaus, and subsequently decreases again (Figure 2c). The observed increase in Δ*F* reflects a decrease in the effective mass on the sensor disk. Since the polymer layer is covalently bound to gold via thiol groups, this mass change is not due to polymer detachment but instead results from changes in layer hydration.^41,42^ Dissipation (Δ*D*) follows a complementary pattern: it decreases rapidly at the start of heating, slows down around 32–34 °C and then exhibits another ramp of decrease as the temperature increases. During cooling, Δ*D* increases steadily back to its initial value. Since Δ*D* reflects the viscoelastic properties of the layer, these changes indicate a change in the density of the polymeric monolayer. This corresponds well to the fact that p­(DEGMA-*co*-OEG_5_MA) hydration is driven by hydrogen bonding between the oligoethylene glycol segments in copolymer substituents and water molecules. It is also well-known that the hydrogen bonding strength is temperature-dependent, becoming weaker as temperature increases. Notably, this process should be gradual as a function of temperature, and it did not explain the nonlinear distribution of curves (Δ*F* and Δ*D*, Figure 2c) during heating and cooling of the polymeric monolayer on QCM-D. To find the exact values of such an extremum, the Δ*F* and Δ*D* curves (presented in Figure 2c) were differentiated, and the first derivative plots are depicted in Figure 2d. As expected, the nonlinearity in the change of resonance frequency (Δ*F*) and dissipation (Δ*D*) of the monolayer is related to the LCST of copolymers. During the heating of the monolayer, the minimum of the derivative Δ*F* curve is 36.6 °C and corresponds well with the determined DLS LCST value of polymers in solution (Figure 1f). During the cooling, the hysteresis of the derivative Δ*F* is observed with a minimum at 38.2 °C. This could have happened because the 10 min equilibration time between temperature changes was insufficient to achieve full hydration/reswelling of the polymeric monolayer. Importantly, the polymer monolayer remained stable throughout the heating–cooling cycle, as both Δ*F* and Δ*D* returned nearly to their initial values, with only minimal deviation. This confirms that the observed changes in mass and viscoelasticity are driven by reversible hydration dynamics rather than detachment of polymer chains from the surface. Additionally, the behavior of p­(DEGMA-*co*-OEG_5_MA) during heating–cooling cycles was analyzed using SE. The collected data were modeled using Complete EASE software, enabling evaluation of layer thickness and surface roughness at various temperatures. Optical modeling was conducted at three distinct temperatures: 20 °C, where the polymer is fully hydrated; 38 °C, which is slightly above the LCST; 50 °C, significantly above the LCST. As anticipated, the thickness of the polymer film decreased with increasing temperature. Specifically, the polymer layer thickness reduced from 20.1 nm at 20 °C to 13.01 nm at 38 °C, and further to 11.08 nm at 50 °C. Comparable results have been reported by other researchers, as pNIPAM polymer brushes exhibited a rapid reduction in polymer layer thickness near the LCST.^21,22^ The observed shrinkage in polymer layer thickness correlates well with the decrease in Δ*D* from QCM-D measurements. Conversely, surface roughness, as determined from optical models, increased from 2.38 nm at 20 °C to 3.04 nm at 50 °C. When the polymer is fully hydrated, the surrounding water makes the upper part of the layer appear smoother than in its collapsed state. At temperatures above the LCST, fewer water molecules are bound to the polymer, resulting in a less uniform layer.

Once the successful formation of the polymer monolayer and its thermoresponsive behavior on QCM-D were confirmed, we evaluated its protein-repellent properties. BSA adsorption was tested at two representative temperatures: 20 °C, where the polymer layer is swollen and hydrated, and 40 °C, where the polymer chains are collapsed (above LCST). For reference, BSA adsorption was first measured on a nonmodified QCM-D sensor (Figure 2e, black curves). At 20 °C, the Δ*F* shift was −13.5 Hz, confirming strong protein adsorption on bare gold.

When the polymer-coated sensor was exposed to BSA under the same conditions, the Δ*F* shift was only −5 Hz (Figure 2e, yellow curve), corresponding to a 63% reduction in BSA adsorption. After rinsing with water and performing a heating–cooling cycle, BSA was injected again (Figure 2e, blue curve). In this case, the Δ*F* change was negligible, indicating that the surface remained effectively protein-repellent, with both the polymer layer and previously adsorbed BSA blocking further adsorption sites. Importantly, an additional BSA injection after 24 h (Figure 2e, red curve) produced no significant Δ*F* shift, confirming the long-term stability of the protein-repellent properties.

An additional experiment was conducted at 40 °C, where the p­(DEGMA-*co*-OEG_5_MA) monolayer on gold was in a collapsed state (above LCST). In this state, the free binding area of the QCM-D sensor disk is fully covered by the polymer chains, providing complete protection against BSA adsorption.^43^ Notably, upon BSA injection, a small increase in Δ*F* was observed (Figure 2f, yellow curve). The displaced water molecules remaining near the surface of the collapsed monolayer can weakly interact with exposed ethylene glycol fragments, forming an interfacial hydration layer. When BSA molecules approach the surface, they displace these interfacial water molecules, which diffuse away, resulting in an apparent mass loss and the observed increase in Δ*F*. Crucially, no direct BSA adsorption occurred, as confirmed by subsequent injections (Figure 2f, blue and red curves), which produced no further frequency changes. For better visualization of the protein-repellent properties of BSA driven by the thermoresponsive monolayer on the QCM-D gold surface at different temperatures, the processes at 20 °C (swollen) and 40 °C (collapsed) are illustrated in Figure 3. These results demonstrate that the polymer monolayer maintains strong protein-repellent properties even above the LCST, under collapsed conditions. This highlights the robustness of the monolayer as a thermoresponsive antifouling surface, expanding its potential applications in biological and biomedical environments.

**Figure 3:**
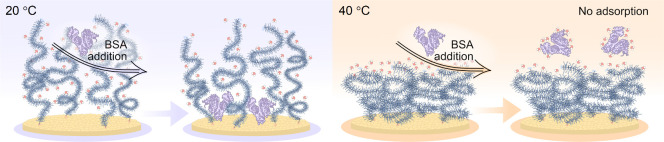
Schematic illustration of BSA–protein repelling by the p­(DEGMA-*co*-OEG_5_MA) monolayer on gold QCM-D surfaces at 20 °C (swollen, hydrated state) and 40 °C (collapsed state above LCST).

To further validate the protein-repelling behavior observed by QCM-D, AFM was used to analyze the surface morphology of both bare and polymer-modified QCM-D sensors before and after exposure to BSA (Figure S10). The bare QCM-D surface displayed a relatively uniform topography with a RMS roughness of 2.072 nm. After polymer coating, the surface became smoother and more homogeneous, as indicated by a reduction in RMS roughness to 1.369 nm, which suggests the formation of a uniform polymer layer. Following exposure of the polymer-coated surface to BSA, led to the appearance of small nanoscale protrusions and a slight increase in surface roughness to 1.572 nm, consistent with limited protein adsorption. In contrast, direct exposure of the unmodified QCM-D surface to BSA produced a markedly different morphology, characterized by pronounced aggregates and a heterogeneous topography, with the RMS roughness increasing to 3.323 nm. These observations align with the QCM-D results and further demonstrate that the polymer monolayer effectively suppresses nonspecific BSA adsorption compared with the bare surface.

## Conclusions

While thermoresponsive surface modifications using polymers such as pNIPAM, pOEGMA, and pDEGMA have been reported previously, to the best of our knowledge, this is the first instance of directly forming hydrophilic p­(DEGMA-*co*-OEG_5_MA) monolayers on gold QCM-D sensors. This approach enabled precise tuning of the LCST and allowed for real-time kinetic monitoring of both monolayer formation and temperature-triggered responses. Therefore, this study serves as an insightful demonstration of the dynamics of monolayer formation and the thermoresponsive behavior of this hydrophilic polymer under thermal stimuli. It also facilitates a deeper understanding of the principles of stimulus-responsive polymers, not only in bulk and solution phases, but also at the monolayer level.

Additionally, this work highlights the significant advantages of controlled polymerization in producing p­(DEGMA-*co*-OEG_5_MA) monolayers, which exhibit outstanding antifouling capabilities and easily adjustable LCST temperature. The ability of these polymer monolayers to effectively repel BSA underscores their promising potential in developing advanced coatings that prevent biological fouling and enhance the biocompatibility of medical devices. Such polymeric layers are crucial for applications such as implantable medical devices, biosensors, and drug delivery systems, as they help minimize immune responses and preserve functionality by preventing protein adhesion to surfaces.

## Supplementary Material



## Notes

^1^H NMR spectra of the p­(OEG_5_MA) homopolymer, including assigned proton chemical shifts, and polymer formation kinetics monitored by SE with the ellipsometry parameter’s Δ evolution as a function of time during polymeric monolayer formation (PDF)

## Abbreviations

^1^H NMR: proton nuclear magnetic resonance spectroscopy
Δ: ellipsometry parameter
Δ*D*: dissipation
Δ*F*: resonance frequency
ACVA: 4,4-azobis­(4-cyanovaleric acid) (thermal initiator)
BSA: bovine serum albumin
CTA: chain transfer agent
DEGMA: di­(ethylene glycol) monomethyl ether methacrylate
DLS: dynamic light scattering
DMSO-*d*_6_: dimethyl sulfoxide (deuterated)
d*n*/d*c*: refractive index increment
DO: 1,4-dioxane
DP: degree of polymerization
LCST: lower critical solution temperature
LS: light scattering
*M*_n_: number-average molecular weight
*M*_w_: weight-average molecular weight
NIPAM: *N*-isopropylacrylamide
OEGMA: oligo­(ethylene glycol) monomethyl ether methacrylate
PEO: poly­(ethylene oxide)
QCM-D: quartz crystal microbalance with dissipation
RAFT: reversible addition–fragmentation chain-transfer (RAFT)
RI: refractive index
SE: spectroscopic ellipsometry
SEC: size-exclusion chromatography
UCST: upper critical solution temperature
