# Supplementary material for: Stimuli-Responsive Poly[oligo(ethylene glycol) methacrylate] Monolayers: Reversible Temperature-Driven Swelling Dynamics, Tunable LCST, and Antifouling Properties
Source: ACS Appl Polym Mater. 2026 May 14;8(11):8456–65. doi: 10.1021/acsapm.6c00888 (PMC13270464; doi:10.1021/acsapm.6c00888)
Supplement: Supplementary file 1 [file ap6c00888_si_001.pdf]

Supporting Information

Stimuli-responsive poly(oligo(ethylene glycol) methacrylate monolayers: reversible temperature-driven swelling dynamics, tunable LCST, and antifouling properties

Silvija Juciute[1], Egle Ezerskyte[1], Kristina Bolgova[1], Medeina Steponaviciute[1], Emile Peciukaityte[3], Ieva Plikusiene[1,4*] and Vaidas Klimkevičius[1,2*]

[1]Institute of Chemistry, Faculty of Chemistry and Geosciences, Vilnius University, Naugarduko str. 24, LT-03225, Vilnius, Lithuania

[2]Biomedical Physics Laboratory, National Cancer Institute, Baublio str. 3b, LT-08406 Vilnius, Lithuania

[3]Institute of Biosciences, Life Sciences Center, Sauletekio al. 7, LT- 10257 Vilnius, Lithuania

[4]State Research Institute Centre for Physical and Technological Sciences, Sauletekio av. 3, LT-10257, Vilnius, Lithuania

Correspondance: vaidas.klimkevicius@chf.vu.lt, ieva.plikusiene@chgf.vu.lt

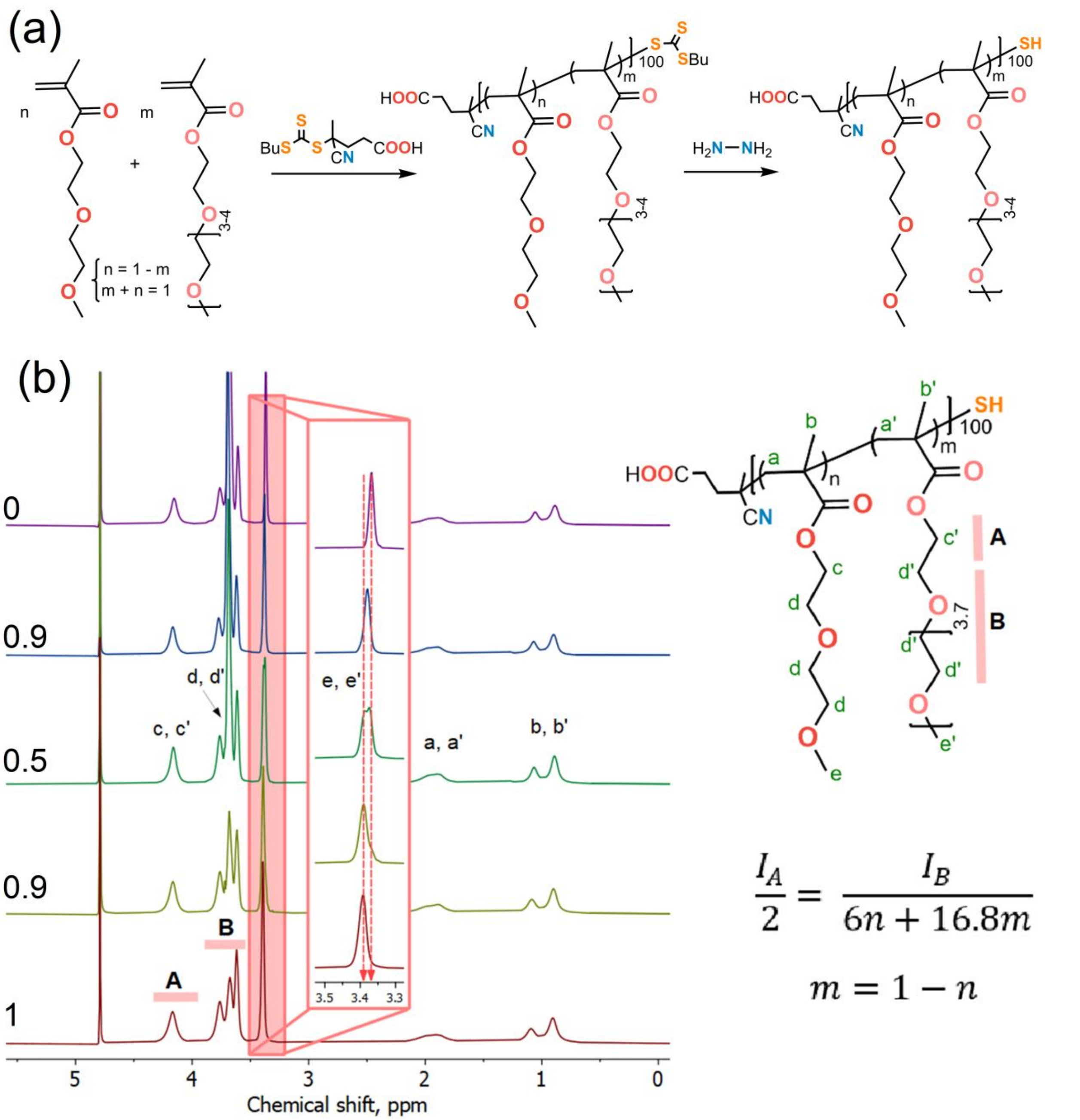

Figure S1. Synthesis and modification of p(DEGMA-*co*-OEG5MA) copolymers with varying compositions (a); ^{1}H NMR analysis of copolymers synthesized using different DEGMA monomer feed ($f_{1(DEGMA)}$), including assigned proton chemical shifts and marked regions integrated for accurate calculation of copolymer composition (b). The magnified zone (inset) indicates the $-OCH_3$ chemical shifts in DEGMA and OEG_5MA substituents.

It is important to note that numerous publications focus on thermo-responsive properties of p(DEGMA-*co*-OEG5MA) copolymers and the tailoring of such properties by varying

the composition in the reaction feed, but not focusing on the evaluation of the exact composition of monomeric units in final products, considering that similar copolymers have the same reactivity, resulting in ideal azeotropic product formation. Although the chemical structures of the monomers DEGMA and OEG_5MA, which contain ethylene glycol-based side chains of varying lengths, are similar, the energy required for the spin exchange of the terminal $-OCH_3$ proton signals differs. In the case of OEG_5MA, the increased flexibility of the longer ethylene glycol side chains results in a higher energy barrier for proton spin exchange. Consequently, the chemical shifts of the $-OCH_3$ groups in the OEG_5MA monomeric units appear at lower ppm values (as shown in the inset of **Figure S1**); however, precise calculations are hindered by overlap with the shifts of DEGMA substituents. The exact polymer composition was determined by comparing the integrals of the oxymethylene group protons' chemical shifts (2H, 4.00-4.35 ppm, marked as I_A) with the total shifts of other methylene protons in DEGMA (6H) and OEG_5MA (average of 16.8H) (3.40-3.90 ppm, marked as I_B). Typically, the total number of methylene protons in OEG_5MA monomeric segments should amount to 20 (2 from the oxymethylene group plus 18); however, this is not entirely accurate. The manufacturer (Sigma Aldrich, Germany) indicated that the average molecular weight of the monomer is 300 g/mol, with a potential variation between 280 and 320 g/mol, indicating a mixture of OEG_4MA and OEG_5MA. The exact composition was experimentally determined from the ^1H NMR spectra of the p(OEG_5MA) homopolymer (**Figure S2**). The calculations revealed that the average length of the ethylene glycol substituent in the OEG_5MA monomeric segment is 4.7. This number was used for further calculations of p(DEGMA-*co*-OEG_5MA) copolymer composition. Copolymers intended for ^1H NMR analysis were selected randomly (see **Table S1**). By using the precise number of protons in the OEG_5MA monomeric units, we found that the composition of the synthesized copolymers closely matched that of the original monomer mixture in the feed. This indicates that the conversion of monomers during the synthesis was nearly complete in all cases.

Table S1. Exact composition of randomly selected p(DEGMA-*co*-OEG_5MA) copolymers calculated from ^1H NMR spectra ($[M]_0:[CTA]_0:[I]_0$ = 300:3:1, 70 °C, 24 hours, DP_{theor} = 100).

**f_1(DEGMA)**	0.9	0.8	0.5	0.1
**F_1(DEGMA)†**	0.909	0.803	0.504	0.104

† - calculated from ^1H NMR spectra

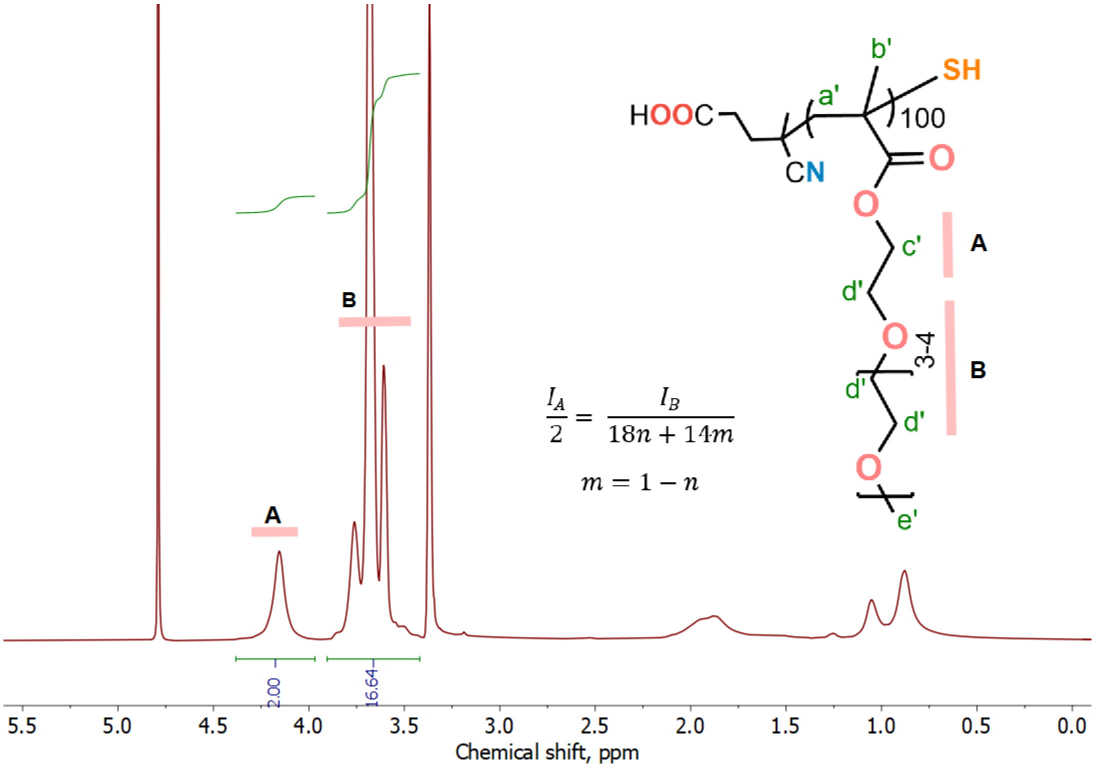

Figure S2. ^{1}H NMR spectra of the p(OEG5MA) homopolymer, including assigned proton chemical shifts and the marked regions integrated for accurate calculation of the substituent length.

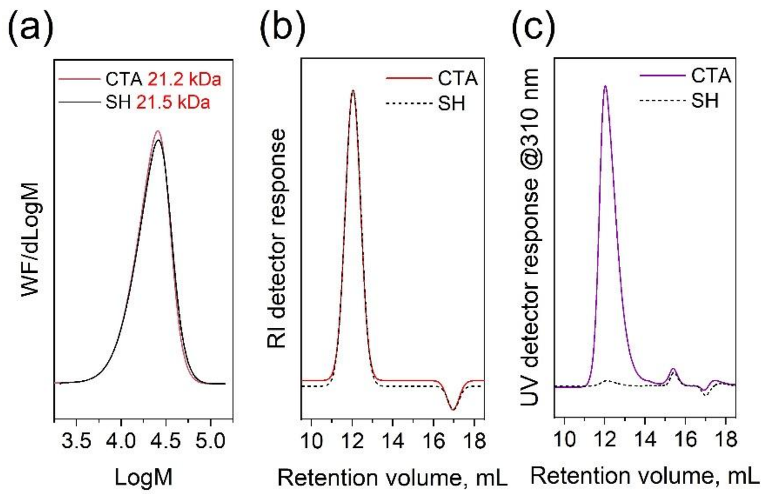

Figure S3. MWD curves (b), SEC-RI (c), and SEC-RI eluograms (d) of p(DEGMA-*co*-OEG5MA) copolymer ($f_{1\ (DEGMA)}$ = 0.8) before and after CTA terminal group removal with hydrazine.

The ^{1}H NMR spectra of -CTA and -SH terminated copolymers are virtually identical, proving that there were no significant changes in composition after aminolysis of terminal CTA groups (**Figure S4**)

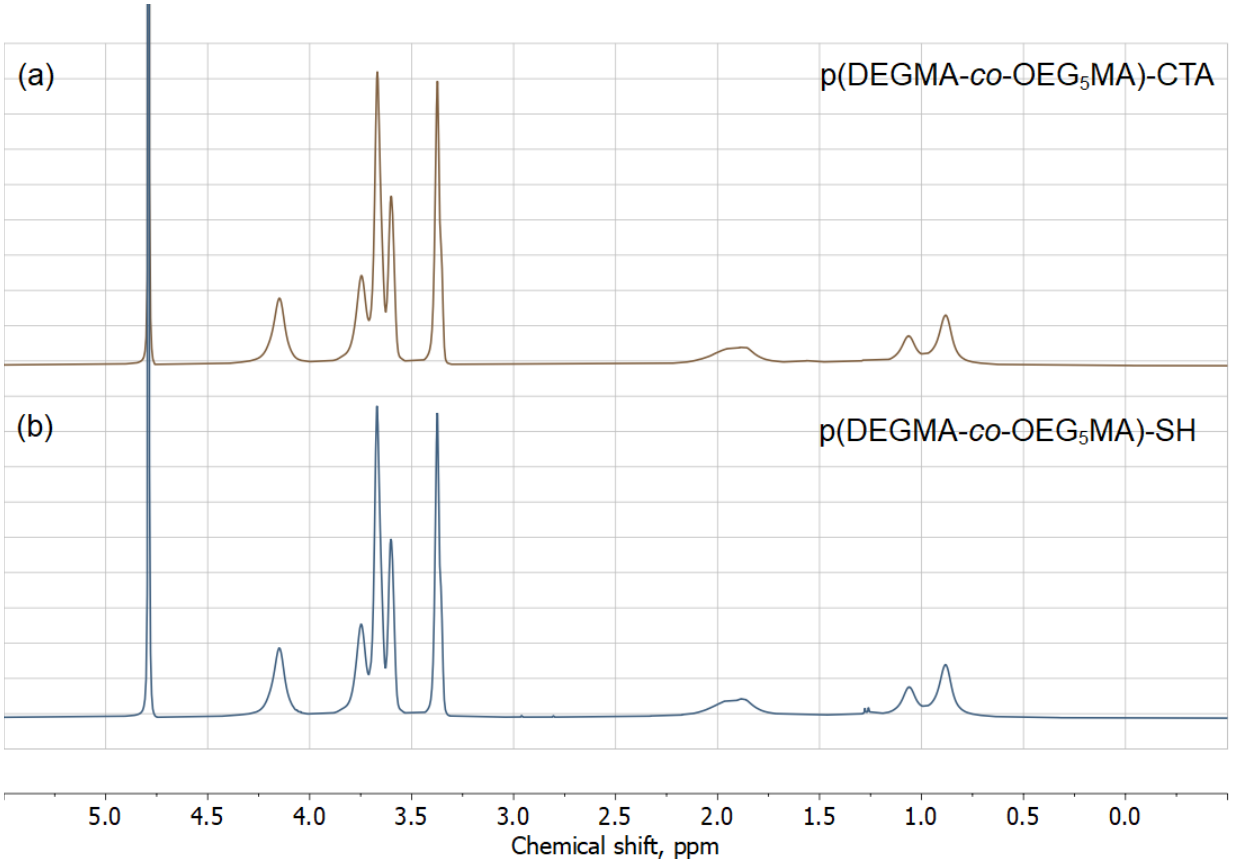

Figure S4. ^{1}H NMR spectra of p(DEGMA-*co*-OEG5MA) copolymers before (a) and after CTA group hydrolysis.

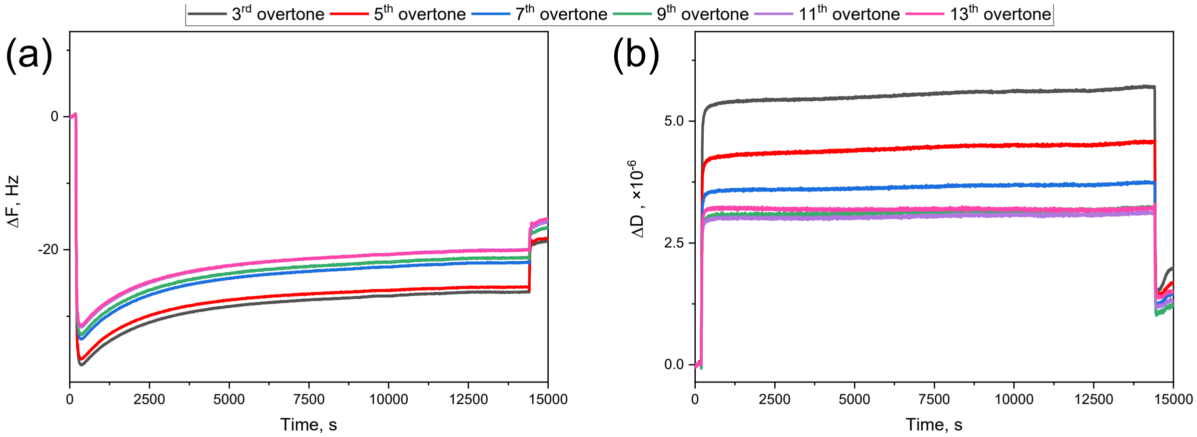

Figure S5. Kinetics of polymer monolayer formation: (a) evolution of resonance frequency (ΔF) and (b) dissipation (ΔD) over time of all measured overtones.

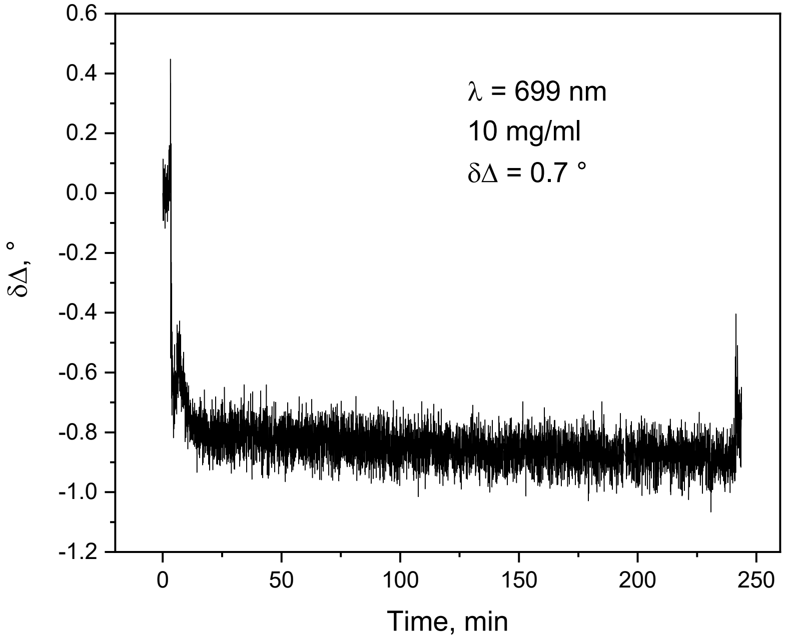

Figure S6. Polymer formation kinetics monitored by SE: ellipsometry parameter's Δ evolution as a function of time during polymeric monolayer formation.

To characterize the formed polymer layer, optical modelling was performed. A layered optical model was applied, with each layer defined by its thickness and effective refractive index. The optical constants of quartz were used to describe the substrate. Layer 1 was modelled as a gold-containing layer using optical constants obtained from the literature, with a thickness of 200 nm as specified by the manufacturer (Biolin

Scientific). For modelling of the polymer layer, the Effective Medium Approximation (EMA) was employed. The EMA model consisted of two components: a polymer fraction and water. The polymer fraction was described using a Cauchy dispersion model with parameters A = 1.460 and B = 0.010. Deionized water was selected as the second component and was also described using a Cauchy dispersion model, with parameters A = 1.299 and B = 0.01030. The thickness of the polymer layer was determined to be 20.10 nm. The volume fraction of water (material 2) within the EMA model was 88.1%. In addition, surface roughness was included as a fitted parameter, yielding a value of 2.38 nm.

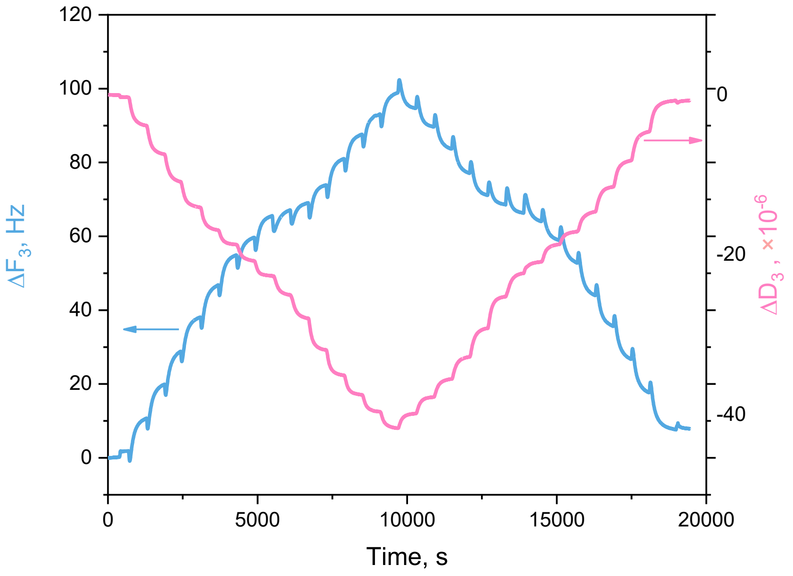

Figure S7. Changes in ΔF and ΔD during a time-dependent heating-cooling cycle. The temperature was increased in 2 °C intervals with an equilibration time of 10 min at each temperature step.

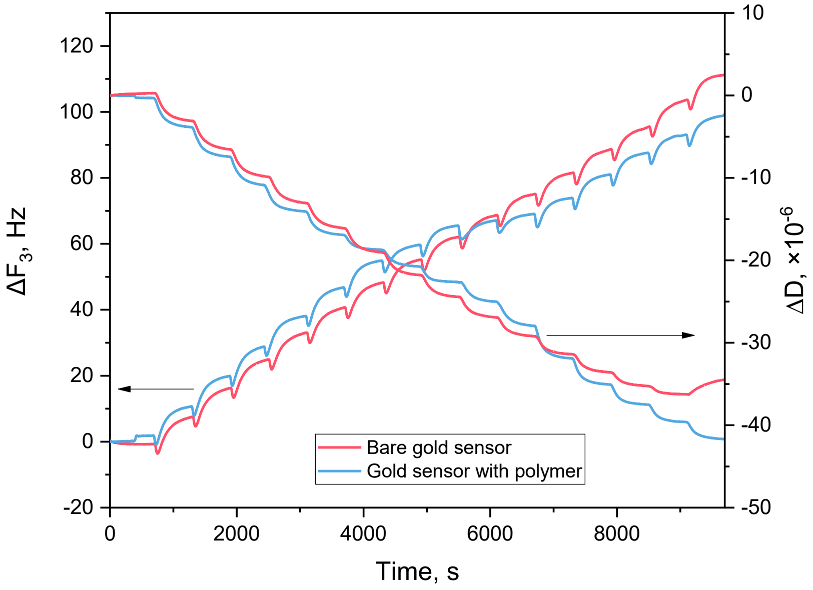

Figure S8. Frequency and dissipation changes in time during the heating cycle for the bare gold sensor (pink curve) and the gold sensor with polymer (blue curve).

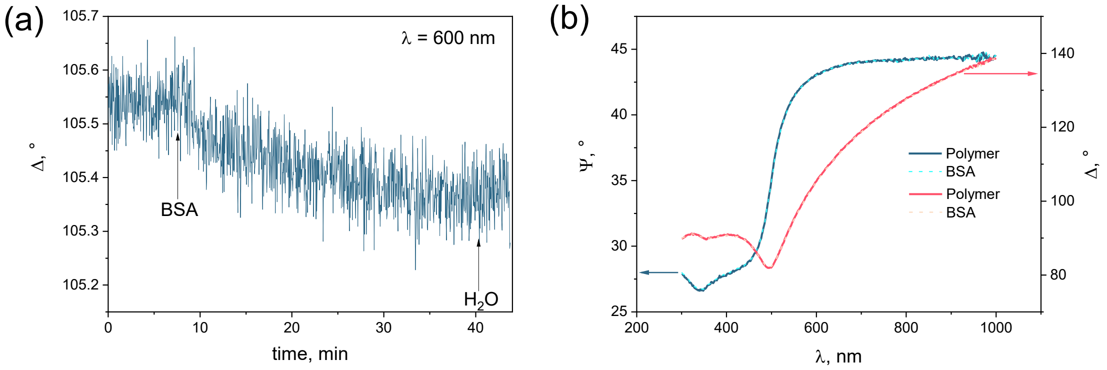

Figure S9. (a) Time-dependent change of the ellipsometric parameter Δ during BSA interaction with the polymer layer. (b) Ellipsometric spectra of Ψ (blue) and Δ (pink) recorded before (solid lines) and after (dashed lines) BSA exposure.

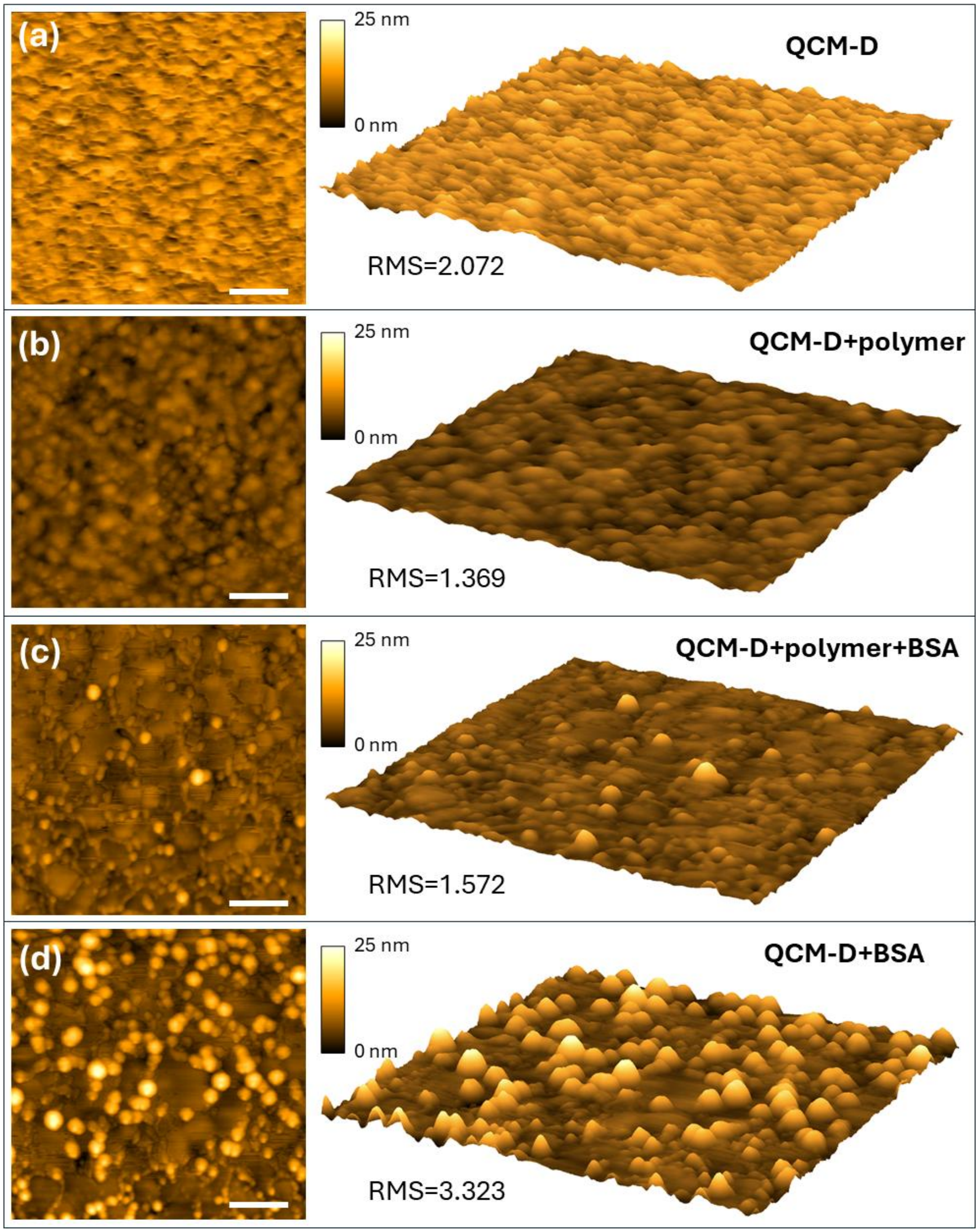

Figure S10. AFM topography images (left) and corresponding 3D renderings (right), together with the extracted RMS roughness values, for QCM-D surfaces: (A) bare QCM-D, (B) QCM-D coated with polymer, (C) QCM-D coated with polymer and exposed to BSA, and (D) QCM-D exposed to BSA. All images were acquired over a scan area of 1 × 1 μm² (scale bar: 200 nm).
